# Longitudinal Gastrectomy for Nonbariatric Indications

**DOI:** 10.1155/2021/9962130

**Published:** 2021-05-06

**Authors:** Oluwatobi O Onafowokan, Aboubakr Khairat, Mohammad Jamal, Hemant Chatrath, Hugo J. R. Bonatti1

**Affiliations:** ^1^University of Maryland Community Medical Group, Easton, MD, USA; ^2^Meritus Health, Hagerstown, MD, USA

## Abstract

**Background:**

Sleeve gastrectomy is the most commonly performed bariatric procedure. Laparoscopic longitudinal gastrectomy (LLG) may be indicated for other indications. *Patients and Methods*. Two men and two women aged 67, 72, 77, and 80 years underwent LLG for nonbariatric indications with two having normal weight, one being cachectic, and one severely obese.

**Results:**

LLG was discussed with patients prior to surgery, but decision for LLG was made during surgery after contemplating other surgical options. A wide sleeve over a 42 French bougie was created with the staple line being oversewn with running 3–0 silk. Indications included a bleeding Dieulafoy lesion that failed endoscopic clipping, fundus gland polyposis found during paraesophageal hernia repair, fundus nodules suspected to be leiomyosarcoma metastases revealing splenosis on final pathology, and significant gastric dilatation associated with organoaxial gastric volvulus. Three patients had an uneventful recovery; the severely obese patient temporarily lost weight but died after two years from a stroke. The last patient developed dysphagia due to an alpha-loop in the sleeve, which was managed by endoscopic stenting. The device subsequently migrated and was laparoscopically removed, with a side-side gastrogastrostomy performed to straighten the alpha-loop. The patient tolerated food better and with overnight PEG tube feeds gained weight but continued heavy smoking. He died after one year from COPD exacerbation.

**Conclusion:**

LLG seems to be an appropriate intervention for various gastric pathologies. Training of residents and fellows in the minimally invasive surgical steps of LLG is encouraged.

## 1. Introduction

Sleeve gastrectomy has emerged as the most commonly performed bariatric procedure during the past decade [1, 2]. The sleeve, vertical, lateral, or longitudinal gastrectomy is not only a bariatric procedure but was suggested to treat duodenogastric biliary reflux as part of the duodenal switch operation [3–6]. The first series were performed using laparotomy, but soon, the laparoscopic approach was adopted to treat morbid obesity [4]. A series of staged duodenal switch operations demonstrated adequate weight loss with a sleeve gastrectomy alone in most patients with moderate morbid obesity (BMI <50 kg/m2). Popularity of sleeve gastrectomy has dramatically increased [7] due to the relatively simple surgical technique, good short- and long-term results, and the low complication rate when compared to Roux-en-Y gastric bypass (RYGBP) and adjustable gastric banding (AGB) [2, 8].

Laparoscopic longitudinal gastrectomy (LLG) and variations of the procedure may also be performed for indications other than weight loss. Resection of the larger curvature to treat tumours in the area such as GIST or adenoma can be performed using a laparoscopic approach [9–11]. Okeny et al. reported an open limited sleeve gastrectomy in a patient with organoaxial volvulus and necrotic fundus [12] with a second look saving the patient a total gastrectomy. LLG has been performed in cases of gastric volvulus within a large hiatal hernia [13], a Bochdalek hernia [14], and associated with AGB [15]. A modification of the procedure has been suggested to treat fundus varices [16], and Collis gastroplasty with fundectomy has been reported as an appropriate lengthening procedure for brachyesophagus [17].

We herein report a series of LLGs performed for non-bariatric indications.

## 2. Patients and Methods

The surgical database of a surgeon with fellowship training in minimal invasive and bariatric surgery was searched for cases of LLG performed for indications other than weight loss between 2015 and 2019 at two rural hospitals. Demographic and clinical data were obtained from the electronic medical records and entered into an EXCEL database.

LLG was performed according to principles previously published, with some modifications according to the intraoperative findings [5, 6, 18, 19].

The study was approved by the local ethic committees.

## 3. Results

Four patients who had a LLG for nonbariatric indications were identified, and their clinical course is described in detail (Table 1). There were two men and two women aged 67, 72, 77, and 80 years. Two patients had normal weight, one was cachectic and one was severely obese.

LLG was considered in all cases during preoperative planning (including Collis gastroplasty in a patient undergoing paraesophageal hernia repair) and was discussed with patients; however, in all cases, decision for LLG was ultimately made during surgery after contemplating other surgical options. LLG was performed over a 44 French bougie creating a wide sleeve, with the staple line being oversewn with running 3–0 silk. Intraoperative endoscopy was performed in three out of the four patients.

**Figure 1:**
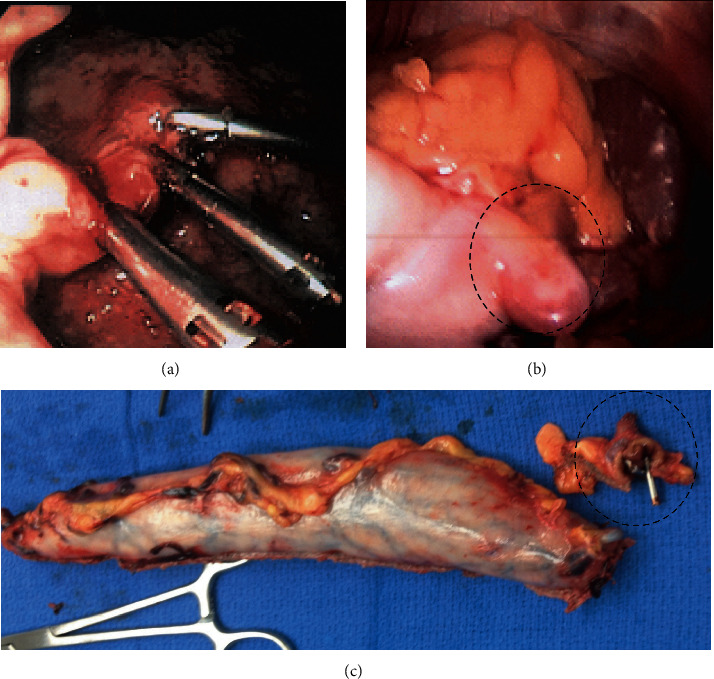
(a) Endoscopy: the bleeding Dieulafoy lesion is clipped. (b) Laparoscopy: a large blood vessel arising from the splenic artery enters the stomach wall. (c) Specimen: sleeve gastrectomy; in the smaller specimen, the endoscopic clips can be seen.

**Figure 2:**
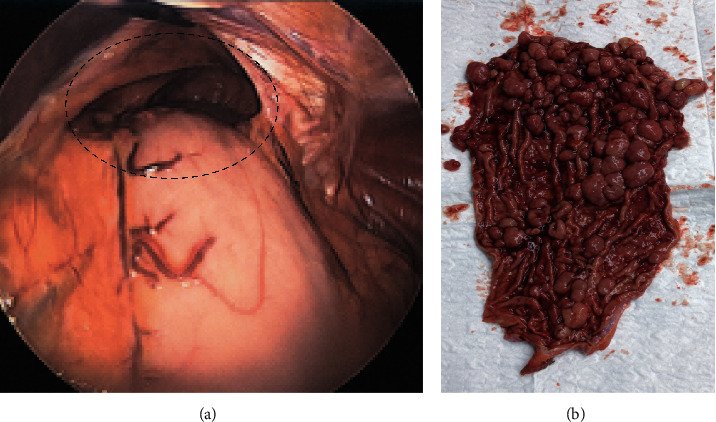
(a) Laparoscopy: large paraesophageal hernia. (b) Opened specimen: fundus gland polyposis.

**Figure 3:**
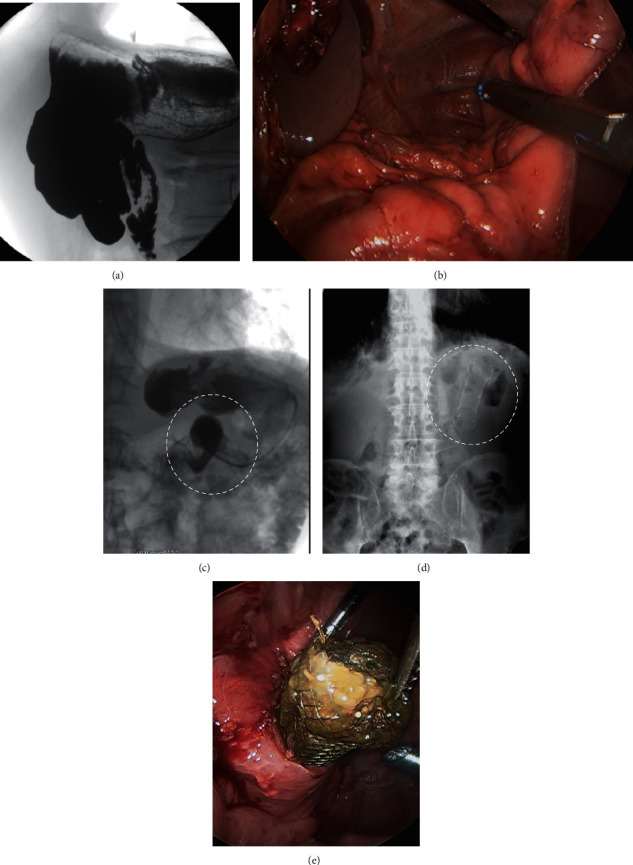
(a) Barium swallow: organoaxial gastric volvulus. (b) Laparoscopy: longitudinal gastrectomy with a very long staple line. (c) Fluoroscopy: alpha-loop in the distal stomach. (d) Abdominal plain film: the stent migrated into the proximal jejunum and is seen in the left upper quadrant. (e) Laparoscopy: the jejunum is opened, and the migrated stent is retrieved.

- *Case 1.* A 75-year-old severely obese female (BMI 35 kg/m^2^) with stage IV renal failure (Cr 2.8 mg/dL), congestive heart failure, and coronary artery disease on clopidogrel (75 mg daily) presented to the emergency room (ER) with haematemesis and melena. She was hypotensive, and hemoglobin was 7 mg/dL. After a fluid bolus and blood transfusions, she stabilised in the ER; proton pump inhibitor infusion was started. She underwent emergent endoscopic clipping of an actively bleeding fundus Dieulafoy lesion (Figure 1(a)). Massive rebleed occurred after 72 hours, and octreotide (50 microgram per hour) was started. On EGD, a large blood clot hindered visualisation. During emergent laparoscopy, the upper portion of the larger curvature of the blood-filled stomach was devascularised. A large blood vessel originating from the splenic artery was clipped (Figure 1(b)), and a LLG was performed. The clipped Dieulafoy lesion was identified in the specimen (Figure 1(c)). The patient had an uneventful postoperative course, her BMI decreased to 26 kg/m^2^, but she was noncompliant with follow-up and refused to come to office visits. She had no reported symptoms from the sleeve gastrectomy but regained weight and died two years later from a stroke.
- *Case 2.* During laparoscopic paraesophageal hernia repair (Figure 2(a)) in an 80-year-old female, the gastric fundus was found to be thickened and immobile making a fundoplication impossible. Intraoperative gastroscopy showed the entire fundus and proximal body occupied by innumerable polyps. After hiatal closure, a wide LLG was performed and a percutaneous endoscopic gastrostomy (PEG) tube was placed in the distal stomach. The specimen showed fundic gland polyposis (Figure 2(b)) which was confirmed on histopathology. Her postoperative course was uneventful. The PEG tube was removed after six weeks, she was followed up annually by her gastroenterologist, had no complications from her sleeve gastrectomy, and maintained a stable weight. The patient remained well after 5 years.
- *Case 3* [20]. A 67-year-old male with a history of metastatic leiomyosarcoma on chemotherapy presented with LUQ pain, and a CT scan showed a mass close to the left adrenal gland. The patient had undergone a trauma splenectomy 40 years ago. During laparoscopic resection of the mass, the stomach was mobilised exposing multiple nodules along the larger curvature, which were suspected to be leiomyosarcoma metastases. The main mass was resected, and thereafter, an LLG was performed. Pathology revealed that the main mass and the gastric implants were splenic tissue that had been growing for extramedullary hemopoesis due to bone marrow toxicity from the chemotherapeutic agents. The patient had an uneventful recovery. He is followed up by oncology, has no complications reported from the sleeve gastrectomy, and has a stable weight. He is alive on chemotherapy for metastatic disease after 4 years.
- *Case 4.* A 72-year-old cachexic male (BMI 14 kg/m^2^), who had a partial colectomy for large bowel perforation >20 years ago, presented with weight loss, worsening dysphagia, and recurrent regurgitation and aspiration for one year. Barium swallow showed organoaxial gastric volvulus (Figure 3(a)). He was a heavy smoker suffering from coronary artery disease and severe chronic obstructive pulmonary disease (COPD). During exploratory laparoscopy, extensive lysis of adhesions exposed a massively distended, atonic stomach. LLG created a relatively long and angled gastric remnant (Figure 3(b)). Postoperative swallow studies indicated delayed oesophageal but prompt transit of dye through the stomach and duodenum. Following discharge on full liquids, he resumed smoking and did not follow dietary recommendations. He returned to the ER after two weeks with dysphagia. Imaging revealed an alpha-loop in the distal stomach (Figure 3(c)) managed by endoscopic stenting. He tolerated a diet and gained weight until stent migration after four weeks (Figure 3(d)). The device was retrieved laparoscopically from the jejunum (Figure 3(e)). By creation of a side-side stapled gastrogastrostomy, the alpha-loop was straightened allowing the endoscope to pass without resistance into the duodenum and a PEG tube was placed. The patient tolerated oral and tube feeds, gained weight, and his quality of life significantly improved. He was followed up by a bariatric surgeon and a nutritionist but remained dependent on tube overnight feeds. He continued smoking and died after one year from COPD exacerbation.

## 4. Discussion

We herein present a heterogenous group of patients who underwent LLG for various indications other than weight loss. This was performed after other options were contemplated and in accordance with previously published cases [9, 11–17, 21–23]. Intraoperative findings ultimately led to decision for LLG. The standards of sleeve gastrectomy were followed according to the fellowship training and adapted to the individual anatomical and pathologic situation [6, 18]. It was aimed to keep the sleeve wider than usual as LLG was not performed for weight loss purposes. An intraoperative endoscopy was performed in three of the four cases.

Outcomes in this series werefavourable. The only obese patient lost weight after LLG but was non-compliant with follow-up. Treatment of acute bleeding from Dieulafoy lesions that failed endoscopic hemostasis may be performed by interventional embolization [24], but surgery is indicated in life-threatening situations with laparosopic fundectomy reported as a viable option [21, 22]. Liu reported a patient who presented with a bleeding Dieulafoy lesion at the lesser curve two days after sleeve gastrectomy, which was managed by endoscopic clipping [25]. Our two patients with benign lesions of the gastric fundus did well, and in both cases, the LLG was part of a more complex surgery including resection of a left upper quadrant mass [20] and paraesophageal hernia repair. In the hiatal hernia patient, removal of the fundus polyposis together with esophageal lengthening was achieved with the LLG [17].

The last patient with gastric volvulus was the most complex case. Sleeve gastrectomy for volvulus has been reported with good outcome [12–14] and, recently, has been shown to be an option to treat gastroparesis [26, 27]. He required a total of seven 60 mm staple loads despite starting well proximal to the pylorus and creating a very long sleeve. As the patient was non-compliant with his diet, a piece of food became lodged in the distal sleeve, causing obstruction and subsequent creation of an alpha-loop. Gastric volvulus, cork-screwing, and kinking following sleeve gastrectomy is a well-known complication [28, 29]. Treatment options include simple observation [30], stenting [28], and conversion to a modified biliopancreatic diversion [31] or RYGBP [29] for early twists. Delayed volvulus of the sleeve may be caused by adhesions between the staple line and liver [32], and lysis of these adhesions may straighten the twist although pexy of the gastric remnant may be necessary and the volvulus may reoccur.

Stenting successfully straightened the gastric twist in our patient, but the device migrated and had to be surgically retrieved. Using a stapled side-side gastrogastrostomy, the twist was straightened, and this may be an alternative to treat similar conditions in patients after sleeve gastrectomy for morbid obesity. Tube feeds through a PEG were well tolerated in our patient showing that the concept of an LLG to resolve the gastric emptying issue associated with the underlying volvulus worked [26, 27]. A gastropexy [33] and/or PEG placement at the 1^st^ operation was contemplated, but would likely have been associated with ongoing inability to empty the gigantic stomach, causing reflux of tube feeds with risk for aspiration, as well as increased intraabdominal pressure pushing the diaphragm up and causing deterioration of ventilation in this patient with severe COPD. After management of the torsion, the patient did well, was able to enjoy some food intake, and gained weight with PEG feed support resulting in significant improvement in his quality of life. Unfortunately, he continued smoking and ultimately succumbed to COPD exacerbation.

To summarize, we confirm that LLG seems to be an appropriate intervention for various gastric pathologies, and the procedure may be used for new additional indications such as gastroparesis [26, 27]. Sleeve gastrectomy is a well-standardized procedure with low complication rates [18]. However, sleeve gastrectomy is not as straightforward as one might think and requires meticulous dissection and a number of operative principles [19]. Complications such as development of an alpha loop, as in one of our patients, or a volvulus may develop. When performing LLG for indications other than morbid obesity, some technical modifications may be necessary. Training of residents in the minimally invasive surgical steps of devascularization of the larger curvature, stapled longitudinal gastric resection over a bougie and hemostasis of the staple line is encouraged.

## Figures and Tables

**Table 1:** Demographic and clinical data.

Age	Gender	Primary surgery	Gastric surgery	Center	Comments	Early complications	Long-term complications	Follow-up
77	f	Bleeding Dieulafoy lesiongastric fundus	NA	LLG, EGD	Obese; had failed endoscopic clipping: recurrent bleed	None	Lost some weight but regained most	Died 2 years later from MI
80	f	Paraesophageal hernia and fundus polyposis	Para esophageal hernia repair	LLG, EGD, and PEG	Could not create fundoplication due to stiff fundus	None	None	Well alive after 5 years
67	m	Nodules LUQ, fundus, liver, and omentum: splenosis on pathology	Removal of the accessory spleen, omentum, and liver biopsy	LLG	History of splenectomy; suspected leiomyosarcoma metastases	None	None	Well alive after 4 years
72	m	Gastric volvulus and intraabdominal adhesions	Extensive lysis of adhesions	LLG and EGD	Cachexia, heavy smoker; esophagus dysmotility; and a very large stomach creating angled sleeve	Nausea for several days; slow emptying of sleeve	Continued smoking; alpha-loop in sleeve: stent; stent migration: relaparoscopy: stent retrieval and gastrogastrostomy; PEG for overnight feeding	Died after one year from COPD

f: female, m: male; NA: not applicable; LLG: laparoscopic longitudinal gastrectomy; EGD: esophagogastroduodenoscopy; MI: myocardial infarction; COPD: chronic obstructive pulmonary disease.

## Data Availability

Data are not available due to HIPAA restrictions.
